# The French General Population’s Perception of New Information and Communication Technologies for Medical Consultations: National Survey

**DOI:** 10.2196/45822

**Published:** 2023-06-16

**Authors:** Rajae Touzani, Elodie Dembele, Emilien Schultz, Alexandra Rouquette, Lorène Seguin, Jean-Charles Dufour, Marie Bannier, Julien Mancini

**Affiliations:** 1 Aix Marseille Univ, INSERM, IRD, ISSPAM, SESSTIM, Sciences Economiques & Sociales de la Santé & Traitement de l’Information Médicale, Equipe CANBIOS Labellisée Ligue 2019 Marseille France; 2 Institut Paoli-Calmettes, SESSTIM U1252 Marseille France; 3 Médialab, Sciences Po, 75007 Paris, France Paris France; 4 Public Health and Epidemiology Department, AP-HP, Bicêtre Hôpitaux Universitaires Paris Sud Le Kremlin-Bicêtre France; 5 Université Paris-Saclay, Univ. Paris-Sud, UVSQ, CESP, INSERM U1018 Villejuif France; 6 Institut Paoli-Calmettes, Département d’Oncologie Médicale Marseille France; 7 APHM, Hop Timone, BioSTIC, Biostatistique et Technologies de l’Information et de la Communication Marseille France; 8 Institut Paoli-Calmettes, Département de Chirurgie Oncologique Marseille France

**Keywords:** new ICT, video recording, mHealth apps, video broadcasting, health literacy, telehealth, teleconsultation, HLS19, COVID-19, France

## Abstract

**Background:**

The development of telehealth and telemedicine, in the form of increased teleconsultation and medical telemonitoring, accelerated during the COVID-19 health crisis in France to ensure continued access to care for the population. Since these new information and communication technologies (ICTs) are diverse and likely to transform how the health care system is organized, there is a need better to understand public attitudes toward them and their relationship with peoples’ current experience of health care.

**Objective:**

This study aimed to determine the French general population’s perception of the usefulness of video recording/broadcasting (VRB) and mobile Health (mHealth) apps for medical consultations in France during the COVID-19 health crisis and the factors associated with this perception.

**Methods:**

Data were collected for 2003 people in 2 waves of an online survey alongside the Health Literacy Survey 2019 (1003 in May 2020 and 1000 in January 2021) based on quota sampling. The survey collected sociodemographic characteristics, health literacy levels, trust in political representatives, and perceived health status. The perceived usefulness of VRB in medical consultations was measured by combining 2 responses concerning this technology for consultations. The perceived usefulness of mHealth apps was measured by combining 2 responses concerning their usefulness for booking doctor appointments and for communicating patient-reported outcomes to doctors.

**Results:**

The majority (1239/2003, 62%) of respondents considered the use of mHealth apps useful, while only 27.6% (551/2003) declared VRB useful. The factors associated with the perceived usefulness of both technologies were younger age (≤ 55 years), trust in political representatives (VRB: adjusted odds ratio [aOR] 1.68, 95% CI 1.31-2.17; mHealth apps: aOR 1.88, 95% CI 1.42-2.48), and higher (sufficient and excellent) health literacy. The period of the beginning of the COVID-19 epidemic, living in an urban area, and being limited in daily activities were also associated with perceiving VRB positively. The perceived usefulness of mHealth apps increased with the level of education. It was also higher in people who had 3 or more consultations with a medical specialist.

**Conclusions:**

There are important differences in attitudes toward new ICTs. Perceived usefulness was lower for VRB than for mHealth apps. Moreover, it decreased after the initial months of the COVID-19 pandemic. There is also the possibility of new inequalities. Hence, despite the potential benefits of VRB and mHealth apps, people with low health literacy considered them to be of little use for their health care, possibly increasing their difficulties in accessing health care in the future. As such, health care providers and policy makers need to consider those perceptions to guarantee that new ICTs are accessible and beneficial to all.

## Introduction

The number of new information and communication technologies (ICTs) is booming. They are increasingly used in medical practices in various ways, including the dematerialization of medical information, telemonitoring and medical assistance, teleconsultation, and electronic patient-reported outcomes (ePROs) [1]. Some new ICTs are now routinely used within the health care system; for example, medical students are provided comprehensive training about synchronous video consultation [2]. Indeed, more than 1 out of 2 French doctors use teleconsultation [3]. However, other new ICTs are still being integrated into the health care system [4]. This is the case for the use of video recording/broadcasting (VRB) of medical consultations and mobile health (mHealth) apps, which both raise new questions regarding their effectiveness, data protection, the digital divide, and acceptability. Because they transform the condition of the patient’s relationship, they have the potential to change the whole experience of care, both in terms of new opportunities and threats. While new ICTs facilitate communication by reducing spatial and temporal constraints [5], there are concerns that they could exacerbate health inequalities on account of various factors, including a lack of universal access and a lack of awareness of users’ level of health literacy [6].

The beginning of telemedicine in France dates to the 1990s [7]. However, it was not until 2009 that the *Hospital, Patients, Health, and Territory* law was put in place to regulate telemedicine practices. According to this law, telemedicine is “a form of remote medical practice using information and communication technologies” [8]. It is therefore a part of telehealth including teleconsultation, tele-expertise, tele-surveillance, tele-assistance, and remote medical first response. Ten years later, the reimbursement of teleconsultation became effective following the social security financing law [9], intending to improve access to care, patient pathways, the quality of prescriptions, and the efficiency of the health care system by facilitating remote consultations. Public policies then promoted the use of teleconsultations through the reimbursement of these consultations at the same rate as in-person consultations, facilitating their adoption.

The COVID-19 pandemic increased the visibility of new ICTs in the context of medical consultations. In France, the first pandemic-related lockdown ran from March 17, 2020, to May 10, 2020. The entire health care system was affected, making it difficult to receive and treat patients with COVID-19 and other diseases. Because of this, French health authorities relaxed the rules regarding the use of new ICTs to facilitate teleconsultations. Accordingly, 80% of general practitioners (GPs) made use of teleconsultations following the update and extension of the rules and conditions for their reimbursement [10]. Following similar trends, rules on mHealth apps were also relaxed for follow-up, contact tracing, and to facilitate digital communication for other types of diseases. Moreover, TousAntiCovid [11], a government app initially designed for contact tracing, extended the visibility of new ICT use in health care for the wider French public. Teleconsultation provided continuity within care pathways, especially for those with chronic illness, and ensured remote monitoring for those who were concerned about going to health care facilities for fear of contracting COVID-19 [12,13].

There has been a wealth of research focused on teleconsultation [14] and its development throughout the COVID-19 pandemic [15,16]. However, features of the teleconsultation technology that could be used to record and broadcast consultations have been understudied. These features were likely more relevant during the health crisis when many hospitals prohibited caregivers and loved ones from accompanying patients. Less research is available on these new practices that have nevertheless proven to be useful for patients, particularly those with low health literacy [17]. Several studies have shown the extent to which telehealth is thought to transform practices, even if it does not fulfill all the promises that were made [7,18]. Given that these new ICTs will potentially concern all the users of the health care system in the future and that they are paving the way for new health care policies, it is important to study their acceptability.

In this context, we focused on those new avatars of telehealth by assessing the perceptions of VRB and mHealth apps for medical consultations in the general population during the COVID-19 health crisis in France and identifying associated factors.

## Methods

### Study Design

Our analyses were based on French data collected in the Health Literacy Survey 2019 (HLS19) of the World Health Organization Action Network project entitled Measuring Population and Organizational Health Literacy (M-POHL) [19]. The first survey wave ran from May 27 to June 5, 2020 [20] and the second from January 8 to 18, 2021. For each wave, a sample of internet users aged 18 to 75 years was drawn from an access panel, reflecting the characteristics of the French general population in terms of gender, age, region, and area of residence. Respondents first had to read an information box about the survey before being able to agree to participate. The internet survey collected information on sociodemographic characteristics (age, level of education), health status indicators, health literacy, ability to navigate the health system, and data related to the COVID-19 pandemic. Respondents’ perceptions of new ICT in the context of medical consultations as well as their confidence in political representatives were also collected.

### Ethics Approval

This study was approved by the Ethics Evaluation Committee of the French National Health and Medical Research Institute (CEEI, IRB 00003888).

### Measures

The perception of new ICT for medical consultations was measured using the following ad hoc question, which focused on 4 different new ICT uses: “In your opinion, how useful are the following means that could be put in place to improve medical consultations? (1) video broadcasting of the consultation over the internet (Skype, Face Time, etc) so that relatives who are not present can participate in the consultation; (2) video recording of consultations to save its content; (3) mobile apps to make and remind people of medical appointments; and (4) mobile apps to send answers to questionnaires evaluating your health to the doctor.” A 4-point Likert scale was used for each response as follows: “not at all useful,” “not very useful,” “quite useful,” and “very useful.” From these, the binary variables were created (0: “not at all useful” and “not very useful,” 1: “quite useful” and “very useful”) for each of the 2 questions on video in consultations. If the participant had a 1 score for either of the 2 questions, they were deemed to perceive VRB as “useful.” The perception of mHealth apps was assessed in the same fashion using the 2 related questions.

A financial deprivation score was calculated based on 3 questions measuring respondents’ ability to (1) pay all their bills at the end of the month (before the COVID-19 pandemic), (2) pay for medication not or partially reimbursed by the health insurance system, and (3) pay for medical examinations and treatments not or partially reimbursed (eg, dental treatment, glasses). For each of these 3 questions, response options were 0 (“very easy”), 1(“easy”), 2 (“difficult”), and 3 (“very difficult”). The deprivation score was computed as the mean of the responses to these 3 questions expressed on a scale from 0 to 100: the higher the number, the greater the financial deprivation [20].

Trust in political representatives was assessed using a binary variable: “very trustworthy” and “quite trustworthy” versus “not very trustworthy” and “not at all trustworthy.”

An annual medical follow-up variable summarized the number of visits to a GP and/or to a specialist into 3 modalities: never, once or twice, and at least 3 times.

The perceived health status indicator was measured by combining responses about the presence or not of chronic morbidity (“Have you had any long-term illness or health problem for at least the last 6 months?”) and about limitations in daily activities (“For at least the past 6 months, how much have your health problems limited the activities you would usually do?”). These 2 questions are the second and third items, respectively, in the Minimum European Health Module (MEHM) [21].

The health literacy level was estimated using the validated French version of the European Health Literacy Survey Questionnaire 12-item (HLS19-Q12-FR) as follows: excellent, sufficient, problematic, and inadequate [20,22].

### Statistical Analyses

Chi-square tests and Student *t* tests were used to compare, respectively, the qualitative and continuous characteristics of respondents according to their perception of each of the 2 new ICTs studied. Multivariate logistic regressions were then used to identify factors associated with a positive perception of each new ICT. After adjustment for age, gender, and survey wave, a backward stepwise procedure was performed to select statistically significant factors in the multivariate models (entry threshold *P*<.20). The Hosmer-Lemeshow test was used to check the fit of the models. To test the robustness of our results, a sensitivity analysis was performed by separately studying the 4 questions on perceptions of ICTs (ie, the 2 questions for each new ICT). The significance level was set to 5% for all statistical analyses. Weighting was applied using “svy” to all commands on Stata software (version 14; StataCorp) to be representative of the French general population in terms of gender, age, region, and place of residence.

## Results

### Description of the Study Sample

Overall, 2003 French adults responded to the survey (including 1003 from the first wave). Half (n=1017, 51.4%) of the participants were women, 69% (n=1369) were under 55 years old, and 77.6% (n=1580) lived in an urban area (Table 1). Just over half had professional activity (n=1235, 61.7%), and the average financial deprivation score was 22.3 (SD 33.7). In terms of GP and specialist consultations, 14.2% (n=281) and 36.3% (n=722), respectively, said they had not had any in the previous 12 months. Slightly less than half (n=900, 44.5%) of the respondents reported having a chronic disease; half (n=452, 22.4%) of these reported being limited in their daily activities. In addition, 83.5% (n=1671) of the respondents did not trust political representatives. Just under half (n=883, 44.1%) declared having limited health literacy, with 14.3% (n=287) reporting inadequate health literacy and 29.8% (n=596) reporting problematic health literacy.

**Figure 1:**
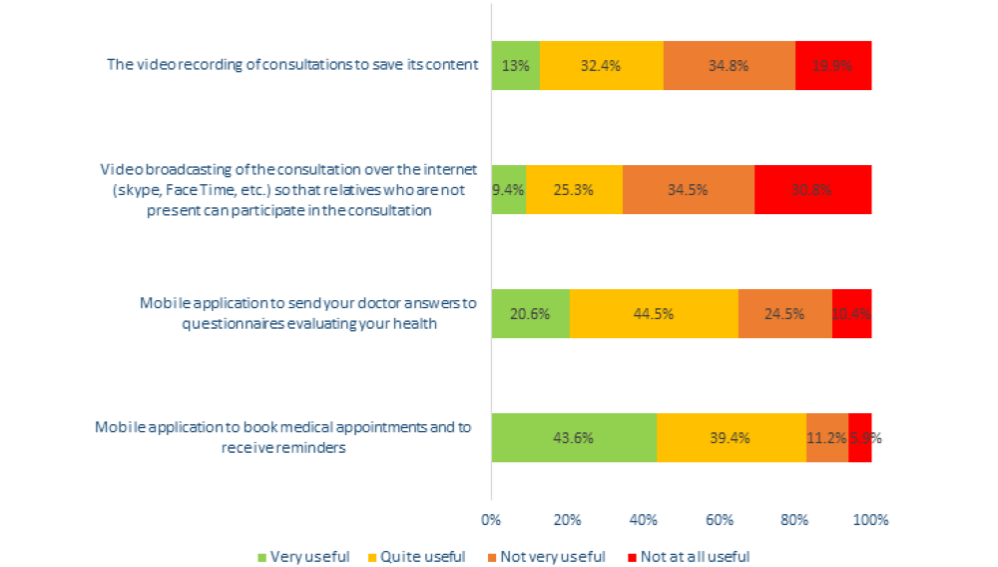
Perception of different new information and communication technologies used to improve medical consultations (N=2003).

Perceived usefulness was higher for mHealth apps than for VRB, especially for the question on medical appointment booking and reminders (Figure 1). After combining answers to both questions for each new ICT, 27.6% (n=551) of respondents were classified as perceiving VRB as useful, while 62% (n=1239) perceived mHealth apps as useful.

**Table 1:** Description of the study population and factors associated with their perception of the usefulness of new information and communication technologies (VRB^a^ and mHealth^b^ apps) in the health care system in France via univariate analysis (N=2003)^c^.

Variables		Total (N=2003)	VRB^a^		*P* value	mHealth apps^b^		*P* value
			Useful (n=551)	Little or not at all useful (n=1452)		Useful (n=1239)	Little or not at all useful (n=765)	
**Survey wave, n (%)**					<.001			.24
	Wave 1	1003 (50.1)	324 (32.3)	679 (67.7)		633 (63.2)	370 (36.8)	
	Wave 2	1000 (49.9)	227 (22.9)	773 (77.1)		606 (60.7)	394 (39.3)	
**Age (years), n (%)**					<.001			<.001
	18-35	594 (30.6)	214 (36.1)	380 (63.9)		416 (70)	178 (30)	
	36-55	775 (38.4)	215 (27.6)	560 (72.4)		488 (62.9)	287 (37.1)	
	56-75	634 (31)	122 (19.3)	512 (80.7)		335 (52.9)	299 (47.1)	
**Gender, n (%)**					.92			.43
	Female	1017 (51.4)	280 (27.5)	737 (72.5)		620 (61.2)	397 (38.8)	
	Male	986 (48.6)	271 (27.7)	715 (72.3)		619 (62.9)	367 (37.1)	
**Country of birth, n (%)**					.32			.96
	France	1914 (95.5)	522 (27.4)	1392 (72.6)		1183 (62)	731 (38)	
	Elsewhere	89 (4.5)	29 (32.3)	60 (67.7)		56 (62.2)	33 (37.8	
**Area of residence, n (%)**					.06			.66
	Rural	423 (22.4)	101 (24.1)	322 (75.9)		257 (61.1)	166 (38.9)	
	Urban	1580 (77.6)	450 (28.7)	1130 (71.3)		982 (62.2)	598 (37.8)	
**Education level, n (%) **					.78			.001
	Primary and lower secondary	358 (17.7)	94 (26.4)	264 (73.6)		192 (53.7)	166 (46.3)	
	Upper secondary	871 (43.7)	239 (27.5)	632 (72.5)		541 (62.1)	330 (37.9)	
	Higher	774 (38.6)	218 (28.4)	556 (71.6)		506 (65.7)	268 (34.3)	
**Professional activity, n (%)**					.002			.001
	Active	1235 (61.7)	370 (30)	865 (70)		799 (64.7)	436 (35.3)	
	Inactive	768 (38.3)	181 (23.7)	587 (76.3)		440 (57.5)	328 (42.5)	
Financial deprivation score (0-100), mean (SD)		22.3 (33.7)	22.7 (33.8)	22.1 (33.7)	.71	21.6 (33)	23.4 (34.8)	.27
**Health status indicator, n (%)**					<.001			.39
	Chronic disease and limited daily activities	452 (22.4)	132 (29.3)	320 (70.7)		294 (65.1)	158 (34.9)	
	Chronic disease and not limited daily activities	448 (22.1)	94 (21)	354 (79)		273 (61.1)	175 (38.9	
	No chronic disease and limited daily activities	102 (5.1)	41 (40.2)	61 (59.8)		58 (57.5)	44 (42.5)	
	No chronic disease and not limited daily activities	1001 (50.4)	284 (28.6)	717 (71.4)		614 (61.4)	387 (38.6)	
**Visits to a GP^d^ in the previous 12 months, n (%)**					.004			.99
	None	281 (14.2)	87 (31.2)	194 (68.8)		172 (61.7)	109 (38.3)	
	1 to 2	798 (39.9)	243 (30.6)	555 (69.4)		497 (62.2)	301 (37.8)	
	3 or more	924 (45.9)	221 (24)	703 (76)		570 (61.9)	354 (38.2)	
**Visits to a specialist doctor in the previous 12 months, n (%)**					.54			.17
	None	722 (36.3)	200 (28)	522 (72)		434 (60.4)	288 (39.6)	
	1 to 2	838 (41.6)	221 (26.4)	617 (73.6)		515 (61.4)	323 (38.6)	
	3 or more	443 (22.1)	130 (29.3)	313 (70.7)		290 (65.7)	153 (34.3)	
**Trust in political representatives, n (%)**					<.001			<.001
	No	1671 (83.5)	415 (24.9)	1256 (75.1)		988 (59.4)	683 (40.6)	
	Yes	332 (16.5)	136 (41.2)	196 (58.8)		251 (75.3)	81 (24.7)	
**Self-reported health literacy level (HLS19-Q12-FR)^e^, n (%)**					<.001			.01
	Inadequate	287 (14.3)	52 (18.1)	235 (81.9)		158 (55.1)	129 (44.9)	
	Problematic	596 (29.8)	125 (21)	471 (79)		357 (60.2)	239 (39.8)	
	Sufficient	795 (39.6)	254 (32.2)	541 (67.8)		507 (63.8)	288 (36.2)	
	Excellent	325 (16.3)	120 (37.1)	205 (62.9)		217 (66.8)	108 (33.2)	

^a^VRB: video recording/broadcasting.

^b^mHealth: mobile health.

^c^Frequency values are crude, and percentages are weighted to be representative of the French population according to gender, age, region, and place of residence.

^d^GP: general practitioner.

^e^HLS19-Q12-FR: French version of the European Health Literacy Survey Questionnaire 12-item.

### Factors Associated With the Perception of Both New ICT for Medical Consultations

Bivariate analyses showed that similar factors were associated with perceiving the 2 studied new ICTs to be useful for medical consultations (Table 1).

Regarding VRB, 32.3% (n=324) and 22.9% (n=227) of respondents from the first and second survey waves, respectively, perceived it to be useful (*P*<.001). The sociodemographic and economic variables associated with this positive perception were younger age (ie, 18-35 years; *P*<.001), having a professional activity (*P*=.002), and living in an urban area (*P*=.06). Some health-related factors were also associated with a positive perception of VRB, specifically having fewer than 3 visits to a GP in the previous 12 months (*P*=.004), not having any chronic disease, and not being limited in one’s usual activities (*P*<.001). Additionally, trust in political representatives and a greater level of health literacy were both associated with a positive perception (*P*<.001).

The sociodemographic and economic variables associated with perceiving mHealth apps to be useful were younger age (*P*<.001), a higher education level (*P*=.001), and being professionally active (*P*=.001). Furthermore, individuals who had a positive perception of mHealth apps reported more than 3 visits to a specialist doctor in the previous 12 months (*P*=.17), trust in political representatives (*P*<.001), and a higher level of health literacy (*P*=.01).

After adjusting for the survey wave, age, and gender (Table 2), the probability of perceiving VRB as useful decreased with time (ie, it was lower in wave 2). Being younger, living in an urban area, trusting political representatives, having a higher level of health literacy, and being limited in daily activities were all associated with perceiving VRB as useful in medical consultations. Professional activity and number of visits to a GP were no longer significant in the adjusted model, but they were associated with age (*P*<.001) and health status indicators (*P*<.001), respectively.

The probability of perceiving mHealth apps as useful increased with the level of education and younger age. Trust in political representatives, having consulted a medical specialist more than once in the previous 12 months, and having a higher level of health literacy were also associated with higher perceived usefulness of mHealth apps. Professional activity was no longer significant in the adjusted model as it was strongly associated with age (*P*<.001) and with the number of visits to a specialist doctor in the previous 12 months (*P*<.001) (Table 2).

The results of the sensitivity analysis (Multimedia Appendix 1) were somewhat similar to those of our multivariate analyses. For VRB, being limited in daily activities, and, to a lesser extent, living in an urban area, were the only 2 variables not significantly associated with perceiving video broadcasting as useful. For mHealth apps, the main difference was that only health literacy was not significantly associated with a positive perception of booking appointments and receiving reminders.

**Table 2:** Factors associated with the perception of the usefulness of new information and communication technologies (VRB^a^ and mHealth^b^ apps) in the health care system in France via multivariate analysis (N=2003).

Characteristics			VRB^c^		mHealth apps^c^
			aOR^d^ (95% CI)		aOR (95% CI)
Survey wave (Ref: wave 1)			0.64 (0.52-0.78)		0.93 (0.77-1.12)
**Age (years)** **(Ref: 56-75)**					
	18-35	2.50 (1.86-3.33)		1.97 (1.54-2.52)	
	36-55	1.57 (1.20-2.06)		1.47 (1.18-1.84)	
Male gender (Ref: female)			1 (0.81-1.23)		1.12 (0.93-1.36)
Urban area of residence (Ref: rural)			1.45 (1.11-1.90)		NC^e^
**Education level** **(Ref: primary and lower secondary)**					
	Upper secondary	NC		1.28 (1-1.66)	
	Higher	NC		1.33 (1.02-1.74)	
**Health status indicators** **(Ref: no chronic disease and not limited in daily activities)**					
	Chronic disease and limited in daily activities	1.30 (1.01-1.69)		NC	
	Chronic disease and not limited in daily activities	0.79 (0.60-1.05)		NC	
	No chronic disease and limited in daily activities	1.82 (1.18-2.82)		NC	
**Number of visits to a specialist doctor in the previous 12 months** **(Ref: none)**					
	1 to 2	NC		1.12 (0.90-1.38)	
	3 or more	NC		1.38 (1.07-1.78)	
Trust in political representatives (Ref: No)			1.68 (1.31-2.17)		1.88 (1.42-2.48)
**Self-reported health literacy level** **(HLS19-Q12-FR^f^) (Ref: inadequate)**					
	Problematic	1.16 (0.80-1.69)		1.22 (0.91-1.63)	
	Sufficient	2.07 (1.46-2.95)		1.41 (1.06-1.86)	
	Excellent	2.44 (1.65-3.61)		1.50 (1.07-2.10)	

^a^VRB: video recording/broadcasting.

^b^mHealth: mobile health.

^c^Ref: little or not at all useful.

^d^aOR: adjusted odds ratio.

^e^NC: not concerned (represents variables not retained in the model with the backward stepwise procedure).

^f^HLS19-Q12-FR: French version of the European Health Literacy Survey Questionnaire 12-item.

## Discussion

### Principal Findings

In this study, we analyzed the perceived usefulness of 2 distinct new ICTs—mHealth apps and VRB—in the context of medical consultations among the French public. Although the level of perceived usefulness was quite different, the factors associated with a positive perception of each one were quite similar.

Overall, 27.6% (n=551) of the study sample considered VRB to be useful in medical consultations compared to 62% (n=1239) for mHealth apps. One possible explanation for this large difference is that respondents may not have considered the use of mHealth apps for making medical appointments or for communicating ePROs to be a new concept. Accordingly, the well-established democratization of smartphones—and thus familiarity with eHealth apps—compared to the more recent use of video in medical consultations could partly explain these different percentages. In addition, the fact that so many new mHealth applications and SMS text messages in health continue to be created reflects their widespread acceptance, which, in turn, suggests they are perceived as useful [23]. Another possible explanation for this difference is that people believe that competent authorities check and control mHealth apps. This perhaps generates a greater sense of trust in using them, unlike the recording or broadcasting of a medical consultation, where competent authorities are considered to be less involved [24]. Another possible explanation for the rather low perceived usefulness of VRB is that people might consider that audio recording/broadcasting is enough for most consultations [25]. It must be mentioned that during the COVID-19 health crisis, with the generalization of telemedicine in many countries worldwide, a high level of patient satisfaction with teleconsultation was reported in France (78%), Israel (89.8%) and India (90%) [26-28]. However, satisfaction with teleconsultation may not systematically translate into perceiving related dimensions like recording and broadcasting to relatives as useful. It is also probable that at the time of the study conducted—after the first few months of the COVID-19 health crisis and after the end of France’s first lockdown—patients wanted direct contact with their doctor and wanted to be accompanied by their relatives. This may have limited their positive perception of VRB. Studies have shown that while many patients find these new practices beneficial, some have reservations, especially regarding the loss of human contact and the fear of inadequately supervised follow-up [29].

In our study, age, trust in political representatives, and health literacy were associated with the perceived usefulness of both VRB and mHealth apps. Young adults’ level of familiarity with new ICT made them more likely than older adults to use these technologies for medical consultations although it was generally beneficial to them [30]. Digital literacy is often lacking in older adults [31], which might explain why the probability of perceiving both studied new ICT as useful decreased with increasing age in our sample. Limited access to digital tools and the internet for this age group can also be a barrier to new ICT use [32], as shown in a study on teleconsultation for postradiotherapy follow-up of patients with prostate cancer over the age of 70 [29]. Findings from a recent study conducted in Germany on barriers to the use of a COVID-19 contact-tracing app [33] also highlighted limited access for older adults. Specifically, the eldest participants (60-77 years old) had poorer access to smartphones and were less able to use the app [33]. The digital divide is a phenomenon first described in the 1990s, which corresponds to the exclusion of part of the population from the use of digital services and devices. Although well characterized, this problem continues due to a lack of effective solutions involving multiple disciplines and different actors [34-36]. In our study, financial barriers to accessing new technologies were not directly highlighted as a significant factor; this suggests that other less visible barriers feed the digital divide (eg, age and health literacy).

People with a higher level of education are more likely to own smartphones and connected devices [37] and more likely to perceive mHealth apps as useful [38,39]. Our findings on the perceived usefulness of mHealth apps echo the association between education and health literacy levels demonstrated in various studies [40,41]. In our study, a higher level of health literacy was associated with a greater probability of perceiving VRB as useful. This implies that a sufficient level of health literacy, and more specifically digital health literacy, could facilitate the use of new ICT in health through greater access, understanding, and use of medical information [6,42]. For respondents closer to the health system, it may also have been the result of a positive novelty bias, either because they were more dependent on medical technology or because they were more inclined to validate new medical advances, as shown in a study on drugs [43]. This is highly problematic, as new ICTs such as VRB can be specifically designed to improve communication in health care for people with low literacy.

We found that respondents residing in an urban area were more likely to perceive VRB as useful. Although one might hypothesize that VRB would be of greater interest to people living in rural areas, it is possible that these people receive more frequent home-based support from a family member and are therefore less inclined to use VRB. Unequal network coverage and disparate availability of digital tools in France might also explain this difference [44]. For example, 87% of people in the Paris metropolitan area have a smartphone compared to 84% in areas with 2000 to 20,000 inhabitants and 77% in rural areas [37]. Besides access to the internet, the use of new ICT is conditioned by many other determinants, including the level of knowledge required to use them properly, the possibility of receiving technical assistance, and the intelligibility of the content. These determinants are all possible sources of differences between users [6,32].

Our analysis also highlighted that trust in political representatives positively influenced perceptions of VRB and mHealth apps. This result is in line with findings from various countries showing that trust in politicians had a positive effect on the intent to use COVID-19 contact-tracing mHealth apps [11,38,45]. Especially regarding health issues, a low level of trust could be a proxy for a critical viewpoint on health policy. This was largely the case for attitude toward the vaccine [46]. In the case of new ICT, specific issues are involved, for instance, the belief of the presence of citizen surveillance and potential breaches of confidentiality, as well as skepticism about the effectiveness of the various technologies used for medical consultations. Alarming claims have been made that new technology, especially 5G devices, now permit panoptic surveillance, including the tracking of all physical movements (geolocation) and internet traffic (cash transactions, online shopping, teleconsultations, etc) [47]. The fact that our study was conducted during the COVID-19 health crisis most probably accentuated these different perceptions. This may explain why the perceived usefulness of VRB decreased in the second wave of the survey, as the level of overall support for government decisions on the management of the pandemic decreased in France over time, especially regarding the COVID-19 health pass, which was introduced linking new technologies to mandatory behaviors [48]. Trust in expertise, both from politics and health authorities, appeared to be affected during the pandemic [49].

Unsurprisingly, medical factors were associated with the perceptions of new ICT in health care in our study. Limitations with daily activities had a significant positive association with perceiving VRB as useful. This may be explained by the fact that these limitations increase the burden (ie, time and cost) of transportation [50-52]. Our finding is consistent with studies elsewhere showing that patients with chronic diseases were satisfied with teleconsultation as an alternative to having to make a physical effort to go to a care center [28,53]. Patients using teleconsultation saved on visit costs and time, while guaranteeing continuity in the health care trajectory by reconfiguring the spatial dimensions of care [54,55]. Similarly, consulting a medical specialist was associated with perceiving mHealth apps as useful. Having repeated medical visits increases the patient’s need to book several appointments and receive reminders. It also increases patients’ interest in communicating remotely with their physicians. One study showed that patients undergoing ambulatory breast reconstruction could be adequately followed via a mHealth app, thereby avoiding the need for face-to-face follow-up visits during the first 30 days after surgery. This remote follow-up was associated with higher patient-reported convenience scores [56].

Our study contributes to the knowledge about the perception of new ICT in French society, in a context where numerous changes in health care took place. In addition, as the frequency of new use of video-related features (recording/broadcasting) in consultation remains unknown, this study provides a first estimate of the perceived usefulness of such new use. Its strengths are the large sample size and the representativeness of the French population obtained by quota sampling. The statistical power was good enough to be able to highlight various factors that influence patients’ perception of new ICT use in health care—results that confirm those already presented in the literature (sociodemographic factors) and other fairly new factors (levels of health literacy, trust in political representatives).

### Limitations

As far as study limitations are concerned, only 4 possible uses of new ICT were examined, 2 regarding VRB and 2 focusing on mHealth apps. In addition, we did not ask for previous use of these ICT and have not given details on the possible use of the videos during the consultation or on their storage (by whom, where, and how?). Moreover, the survey questionnaire was administered via the internet, so the study population had a higher education and was likely to be more familiar with new ICTs. Therefore, our results may overestimate the general public’s perceived usefulness of these technologies. Another limitation is that we did not directly measure smartphone use or digital health literacy or indeed the individual reasons for reluctance to the new ICT studied. Finally, trust in French political representatives was particularly low when the survey was conducted, with a possible influence on the strength of its association with the perceptions of VRB and mHealth apps for medical consultations. Future related work could include more in-depth questions on behaviors regarding the use of new ICTs and patient perceptions of them. An ethnographic study on practices needs to be conducted to complete these results and provide more detailed answers on perceptions, reluctance, and current uses of these new ICTs.

### Conclusions

The integration of new ICTs in medical care is already a reality, but the perception of their usefulness differs according to age, health literacy, and attitude toward institutions. In our study, perceptions of new ICTs differed by technology, pointing to the need to adapt the analysis to specific technology and not consider them as unified. Because their use is going to increase in everyone’s care pathways, there is a need to better understand the differences between individuals, especially to detect the difficulty of use. Despite the help that VRB can provide, particularly for people with poor health literacy, the fact that the latter did not consider this technology as useful could lead to fewer opportunities to improve communication. As the use of new ICT will only increase in the coming years, structural inequalities must be overcome to promote the equitable use of new ICTs in the field of health.

## Appendix

Multimedia Appendix 1 Sensitivity analyses.

## Abbreviations

ePRO: electronic patient-reported outcome
GP: general practitioner
HLS19: Health Literacy Survey 2019
HLS19-Q12-FR: French version of the European Health Literacy Survey Questionnaire 12-item
ICT: information and communication technology
MEHM: Minimum European Health Module
mHealth: mobile health
M-POHL: Measuring Population and Organizational Health Literacy
VRB: video recording/broadcasting

## Data Availability

The data sets generated and/or analyzed during this study are available from the corresponding author upon reasonable request.
