# Supplementary material for: The French General Population’s Perception of New Information and Communication Technologies for Medical Consultations: National Survey
Source: J Med Internet Res. 2023 Jun 16;25:e45822. doi: 10.2196/45822 (PMC10337435; doi:10.2196/45822)
Supplement: Multimedia Appendix 1 [file jmir_v25i1e45822_app1.docx]

**Multimedia Appendix 1**

**Table A1: Factors associated with patient-perceived usefulness of video recording and broadcasting (VRB) in medical consultations in France: multivariate analysis (N=2 003)**

|  | **Video broadcasting^*^** | | **Video recording^**^** | |
| --- | --- | --- | --- | --- |
|  | **aOR** | **95% CI** | **aOR** | **95% CI** |
| **Survey wave** *(Ref :Wave 1)* |  | |  | |
| Wave 2 | 0.69 | 0.55-0.81 | 0.80 | 0.67-0.96 |
| **Age (years)** *(Ref : 56-75)* |  |  |  |  |
| 18-35 | 2.87 | 2.19-3.75 | 1.75 | 1.37-2.24 |
| 36-55 | 1.67 | 1.30-2.14 | 1.41 | 1.13-1.76 |
| **Gender** *(Ref : Women)* |  | |  | |
| Men | 1.04 | 0.85-1.26 | 0.90 | 0.75-1.08 |
| **Area of residence** *(Ref : Rural)* |  |  |  |  |
| Urban | 1.24 | 0.97- 1.59 | 1.30 | 1.03- 1.63 |
| **Health status indicators** *(Ref : No chronic disease and not limited in daily activities)* |  | |  | |
| Chronic disease and limited in daily activities | 1.17 | 0.91-1.50 | 1.45 | 1.15-1.83 |
| Chronic disease and not limited in daily activities | 0.82 | 0.63- 1.06 | 0.96 | 0.76- 1.22 |
| No chronic disease and limited in daily activities | 1.48 | 0.96-2.27 | 2.01 | 1.33-3.05 |
| **Trust in political representatives** *(Ref : No)* |  | |  | |
| Yes | 1.57 | 1.23-2.01 | 1.35 | 1.06-1.72 |
| **Self-reported health literacy level *(HLS_19_-Q12-FR***) *(Ref : Inadequate)* |  | |  | |
| Problematic | 1.46 | 1.04-2.07 | 1.20 | 0.89-1.62 |
| Sufficient | 2.05 | 1.48-2.84 | 1.48 | 1.11-1.97 |
| Excellent | 2.18 | 1.51-3.15 | 2.53 | 1.10-2.14 |

* Ref (Little or not at all useful)

** Ref (Little or not at all useful)

aOR: Adjusted Odds Ratios

95% CI: 95% Confidence Intervals

**Table A2: Factors associated with patient-perceived usefulness of mobile health applications (mHealth apps) in medical consultations in France: multivariate analysis (N=2 003)**

|  | **Booking appointments and receiving reminders*** | | **Communicating ePRO to physician**** | |  |  |
| --- | --- | --- | --- | --- | --- | --- |
|  | **aOR** | **95% CI** | **aOR** | **95% CI** |  |  |
| **Survey wave** *(Ref :Wave 1)* |  | |  | |  |  |
| Wave 2 | 1.01 | 0.79-1.28 | 0.93 | 0.77-1.12 |  |  |
| **Age (years)** *(Ref : 56-75)* |  | |  | |  |  |
| 18-35 | **1.58** | 1.14-2.19 | 2.12 | 1.65-2.74 |  |  |
| 36-55 | 1.25 | 0.95-1.65 | 1.44 | 1.15-1.80 |  |  |
| **Gender** *(Ref : Women)* |  | |  | |  |  |
| Men | 0.89 | 0.70-1.13 | 1.14 | 0.94-1.39 |  |  |
| **Education level** *(Ref : Primary and lower secondary)* | | | | |  |  |
| Upper-secondary | 1.02 | 0.75-1.41 | 1.36 | 1.05-1.76 |  |  |
| Higher | 1.47 | 1.04-2.06 | 1.31 | 1.00-1.71 |  |  |
| **Number of visits to a specialist doctor in the last 12 months** *(Ref : Never)* | | | | |  |  |
| 1 to 2 times | 1.31 | 1.00-1.71 | 1.13 | 0.91-1.40 |  |  |
| At least 3 times | 1.34 | 0.97-1.86 | 1.36 | 1.05-1.77 |  |  |
| **Trust in political representatives** *(Ref : No)* | | | | |  |  |
| Yes | 1.91 | 1.28-2.83 | 1.92 | 1.43-2.57 |  |  |
| **Health literacy level *(HLS_19_-Q12-FR)*** *(Ref : Inadequate)* |  |  |  |  |  |  |
| Problematic | 1.12 | 0.78-1.61 | 1.42 | 1.05-1.91 |  |  |
| Sufficient | 1.23 | 0.87-1.74 | 1.36 | 1.03-1.81 |  |  |
| Excellent | 1.52 | 0.98-2.36 | 1.40 | 1.07-2.10 |  |  |

* Ref (Little or not at all useful)

** Ref (Little or not at all useful)

aOR: Adjusted Odds Ratios

95% CI: 95% Confidence Intervals
